# Isolation and characterization of a native strain of the entomopathogenic fungus *Beauveria bassiana* for the control of the palm weevil *Dynamis borassi* (Coleoptera: Curculionidae) in the neotropics

**DOI:** 10.1007/s11274-024-04044-5

**Published:** 2024-07-05

**Authors:** Yeisson Gutiérrez, Karen A. Alarcón, Cristian Ortiz, Jenny M. Santos-Holguín, Jennifer L. García-Riaño, Cindy Mejía, Carol V. Amaya, Liz Uribe-Gutiérrez

**Affiliations:** 1 Corporación Colombiana de Investigación Agropecuaria–Agrosavia. Centro de Investigación La Libertad, Km. 17 Vía Puerto López, Villavicencio–Meta, Colombia; 2 Corporación Colombiana de Investigación Agropecuaria–Agrosavia. Centro de Investigación El Mira, Km. 38, Vía Tumaco–Pasto, Tumaco–Nariño, Colombia; 3 Corporación Colombiana de Investigación Agropecuaria–Agrosavia. Centro de Investigación Tibaitatá, Sede Tunja–Boyacá, Colombia; 4 Corporación Colombiana de Investigación Agropecuaria–Agrosavia, Centro de Investigación Tibaitatá, Km 14 Vía Bogotá–Mosquera, Mosquera, Colombia

**Keywords:** Biological control, Entomopathogenic fungi, Metabolic profiling, Mass production, Integrated pest management, Neotropical agriculture

## Abstract

This study aimed to isolate and characterize a native strain of *Beauveria bassiana*, coded as Bv065, showcasing its potential as a biological control agent targeting the palm weevil *Dynamis borassi*. Originating from a naturally infected *D. borassi* specimen collected in southwestern Colombia, the fungus underwent molecular identification and was identified as *B. bassiana*, exhibiting high sequence similarity with known reference strains. The physiological characterization revealed that Bv065 thrived within a temperature range of 25 to 30 °C and a pH range of 6 to 9. Moreover, the key carbon sources that allow optimal growth of the strain were identified through metabolic profiling, including sucrose, D-mannose, and γ-amino-butyric acid. These findings offer strategic insights for scalability and formulation methodologies. Additionally, enzymatic analyses unveiled robust protease activity within Bv065, crucial for catalysing insect cuticle degradation and facilitating host penetration, thus accentuating its entomopathogenic potential. Subsequent evaluations exposed Bv065’s pathogenicity against *D. borassi*, causing significant mortality within nine days of exposure, albeit exhibiting limited effectiveness against *Rhynchophorus palmarum*. This study underscores the importance of understanding optimal growth conditions and metabolic preferences of *B. bassiana* strains for developing effective biopesticides. The findings suggest Bv065 as a promising candidate for integrated pest management strategies in neotropical regions, particularly for controlling palm weevil infestations in coconut and peach palm cultivation. Future research avenues include refining mass production methodologies, formulating novel delivery systems, and conducting comprehensive field efficacy trials to unlock the full potential of Bv065 in fostering sustainable pest management practices. Overall, this study contributes to the growing body of knowledge on entomopathogenic fungi and their pivotal role in biological control, offering nuanced perspectives on eco-friendly alternatives to conventional insecticidal interventions.

**Supplementary Information:**

The online version contains supplementary material available at 10.1007/s11274-024-04044-5.

## Introduction

The palm weevils, *D. borassi* and *R. palmarum*, have emerged as significant phytosanitary concerns in the Colombian Pacific region, particularly affecting peach palm (*Bactris gasipaes* Kunth) and coconut (*Cocos nucifera* L.) cultivation (Gaviria et al. 2021; Gutiérrez et al. 2023). These insects’ larvae infest the palm’s stem and apical meristem, causing palm crown collapse and acting as vectors for diseases like red ring disease (Gerber and Giblin-Davis 1990; Oehlschlager et al. 2002). The identification of these species as pests began around 2010, initially attributing damages to the *R. palmarum* insect (Bautista-Giraldo et al. 2020). Further research confirmed *D. borassi* as the primary cause of inflorescence damage and palm decapitation in disturbed forests around Buenaventura, Colombia, resulting in damage of over 50% of inflorescences, which exhibited perforations at the apex, central, and basal regions (Bautista-Giraldo et al. 2020; Gaviria et al. 2021). These damages documented on palms of economic interest echoes symptoms first reported in 1998 on native palms (Couturier et al. 1998). Between 2015 and 2020, the onslaught of palm weevil attacks in Colombia had a substantial negative impact on peach palm and coconut yields (Agronet 2020). This impact is particularly noteworthy because, despite efforts to expand cultivation, the production of these crops in Colombia still falls significantly short of the growing demand. Since 2020, two major phytosanitary issues further exacerbated the situation, resulting in a noticeable decline in productivity and extensive damage, with palm weevils causing up to 70% damage, notably along the Pacific coast (Vásquez-Ordóñez et al. 2020; Gaviria et al. 2021).

Globally, traditional methods for managing palm pests encompass a range of practices, including maintaining overall plant and field sanitation, applying preventive chemical treatments to palm wounds, introducing insecticide-sand mixtures into young palm leaf axils, using curative chemical treatments on infested palms, incorporating biopesticides, and, as a last resort, removing heavily infested palms and incinerating them (Abbas 2010; Hussain et al. 2013; Faleiro et al. 2019). In Colombia, the recommended strategy for palm weevil management leans heavily on cultural practices, including mass trapping, which entices adult weevils using aggregation pheromones (Oehlschlager et al. 2002; Gutiérrez et al. 2023), eliminating breeding sites and removing affected palms (Alvarado et al. 2013; Oehlschlager 2016). Nevertheless, these approaches frequently fall short given the severity of the phytosanitary issue (Ahmed and Freed 2021; Gutiérrez et al. 2023). Moreover, the use of highly toxic synthetic insecticides by palm producers has been observed, an aspect not extensively investigated in Colombia, where concerns about the development of physiological and behavioral resistance within palm weevil populations are a distinct possibility (Gutiérrez 2020; Ahmed and Freed 2021). Additionally, the application of synthetic insecticides with little or no guidance may have significant implications for human and environmental health (Isman 2006; Devine and Furlong 2007).

An environmentally sustainable alternative for palm weevil control is the implementation of biological control via the use of entomopathogenic fungi. In the realm of integrated pest management, entomopathogenic fungi serve as pivotal biological control agents, particularly in tropical agricultural settings (McGuire and Northfield 2020). Notably, *Beauveria* and *Metarhizium* species have demonstrated their effectiveness as biological control agents against a range of agriculturally significant pests, including ticks (Sullivan et al. 2022a), whiteflies (Barra-Bucarei et al. 2020; Sani et al. 2020), citrus psyllids (Maluta et al. 2022) and red palm weevils (Yang et al. 2023). Their broad-spectrum efficacy encompasses all developmental stages of these pests, making them essential components of integrated pest management strategies. Their mechanisms of action involve outcompeting pathogens for nutrients and space while also altering growth conditions. Additionally, entomopathogenic fungi display cryptic behavior within plants, allowing them to establish themselves within plant tissues and maintain their pathogenicity characteristics (Akello et al. 2010). Remarkably, these fungi may even confer stress resistance proteins to the host plant (Gomez-Vidal et al. 2006). Furthermore, they provide protection against larval feeding, with their effectiveness extending for up to three months following inoculation (Ricaño et al. 2013).

*B. bassiana*, a naturally occurring soil-borne fungus belonging to the Hypocreales order and Clavicipitaceae family, is highly regarded for its entomopathogenic capabilities. It possesses the remarkable ability to infect and eliminate a diverse range of insect species, making it a top choice for pest control (Wahengbam et al. 2021). What sets *B. bassiana* apart is its inherent safety for humans, domestic animals, and the environment. This fungus poses no threat to non-target species and leaves no harmful residues in soil, water, or on crops (Meyling et al. 2009; Xiao et al. 2012). Numerous promising applications for sustainable and effective population control have emerged in the utilization of *B. bassiana* against palm weevils. Extensive research conducted in laboratory, semi-field, and field settings consistently demonstrates the pathogenicity of *B. bassiana* towards both larvae and adults of various *Rhynchophorus* species, resulting in significant mortality rates (Dembilio et al. 2010; Lo Verde et al. 2014; León Martínez et al. 2019; López-Luján et al. 2022). Furthermore, *B. bassiana* infections have been shown to reduce egg fertility by approximately 52%, with outcomes influenced by the sex of the treated parent (Dembilio et al. 2010). Exposure of *R. ferrugineus* larvae to *B. bassiana* can also impact larval development, leading to increased energy expenditure in defense against the microbial agent (Hussain et al. 2015). Notably, *B. bassiana* emits volatile organic compounds that function as repellents against the *R. ferrugineus* beetle while concurrently exerting a neurotoxic effect (Jalinas et al. 2022). Additionally, in the realm of population control, infected adult palm weevils, typically surviving for several days, partake in horizontal transmission by dispersing conidia from their cuticles to infect new insects (Dembilio et al. 2018; León-Martínez et al. 2019). Lastly, transgenerational effects have been observed, where the impact of *B. bassiana* extends to the survival rates of larvae descended from inoculated adults, signaling a broader ecological influence (Lo Verde et al. 2014).

The aforementioned attributes collectively establish *B. bassiana* as an excellent candidate for controlling pest palm weevils. However, despite significant advances in the development of fungal biopesticides over the past two decades, the availability of commercial products for their use in controlling agriculturally significant pests remains limited. In Colombia, this constraint is underscored by the absence of registered biopesticides utilizing entomopathogenic fungi, where the only available products, exceeding ten in number, are hormone-based (Sullivan et al. 2022b). Additionally, there is a conspicuous absence of studies to date assessing the pathogenicity of entomopathogenic fungi against the palm weevil *D. borassi*.

The objective of this study was to comprehensively examine a native strain of the entomopathogenic fungus *B. bassiana* (Bv065), encompassing its molecular identification, growth characteristics, metabolic and enzymatic profiles, and its pathogenicity against two weevil species, *D. borassi* and *R. palmarum*. We postulated that the native *B. bassiana* strain (Bv065), isolated from an infected *D. borassi* specimen, would exhibit the attributes required for efficient pest control. Furthermore, our hypothesis included that this strain’s pathogenicity against *D. borassi* and *R. palmarum* weevils would rival, or potentially surpass, that of commercially available bioinsecticides. Additionally, through an in-depth analysis of the culturing conditions, and metabolic and enzymatic profile, we aimed to gather the foundational data necessary for the formulation of a bioinsecticide based on the studied *B. bassiana* strain.

## Materials and methods

### Fungal collection, isolation, and molecular identification

A native strain of the entomopathogenic fungus *B. bassiana* was isolated from an infected *D. borassi* individual found in an experimental peach-palm plantation at El Mira Research Centre in Tumaco, Colombia (coordinates: 78.70072 N, 1.55122 W). This location has an average temperature of 25.8 °C and an average annual precipitation of 3000 mm (IDEAM, 2022).

To promote sporulation on the insect’s surface, the infected insect was placed in a humid chamber at 25 °C for five days. Conidia samples were collected using a sterile entomological needle and inoculated onto potato dextrose agar (PDA Sigma-Aldrich, Darmstadt, Germany) supplemented with 0.1% w/v chloramphenicol antibiotic (Colmed International, Bogotá). The plates were then incubated for ten days at 25 °C until fungal growth became evident. Subsequently, several consecutive inoculations were made on PDA to isolate the fungi using the puncture method. Agar discs containing the fungi were preserved in a solution of 10% v/v glycerol (Sigma-Aldrich, Germany) and 0.1% Universal peptone (Merkc, Burlington Massachusetts, USA) v/w, and stored at − 80 °C. The isolate was registered in the Colombian Corporation of Agricultural Research’s (Agrosavia) Microorganisms Germplasm Bank (Mosquera, Colombia) RCN:129) and assigned the code Bv065. The collection of both, the insect and the fungi, was conducted under collecting permit 1466 of 2014 granted by the ANLA (Autoridad Nacional de Licencias Ambientales) to Agrosavia.

For molecular identification, total DNA was extracted using Zymo Research Fungal/bacterial DNA miniprep TM kit (Zymo Research, California, USA) following the manufacturer’s instructions. Partial amplification of the internal transcribed spacer region (ITS) and the elongation factor gene *TEF1-α* were amplified by polymerase chain reaction using the primers sets ITS1 (5ʹ-TCCGTAGGTGAACCTGCGG-3′) and ITS4 (5ʹ-TCCTCCGCTTATTGATATGC-3ʹ), and EF1-728F (5ʹ-CATCGAGAAGTTCGAGAAGG-3ʹ) and EF1-986R (5ʹ-TACTTGAAGGAACCCTTACC-3ʹ), respectively. The sequencing of amplified DNA fragments was conducted by Corpogen Corporation (https://www.corpogen.org/, Bogotá. Colombia). Ambiguous bases in the forward and reverse trace files were resolved by manually checking against the PHRED scores. The taxonomic identification of the isolate was carried out using the Mycobank and NCBI GenBank databases (https://www.mycobank.org; https://blast.ncbi.nlm.nih.gov) and the basic local alignment search analysis tool (BLASTn). The partial sequences of the ITS region and EF were deposited in GenBank with accession numbers OR135359 and OR136511, respectively.

### Effect of temperature and pH on the growth of *B. bassiana* Bv065

We initiated pure cultures of *B. bassiana* Bv065 in PDA, incubating them at a consistent temperature of 25 °C for 10 days. Following this, we prepared a conidial suspension with a concentration of 1xx10^6^ conidia mL^−1^ in a 0.1% v/v Tween 80^®^ solution (Scharlau, Barcelona).

To evaluate the impact of different temperature conditions on the growth of *B. bassiana* Bv065, we initiated an experiment by inoculating PDA plates with a 2 µL conidia suspension. These plates were incubated for 15 days, spanning a temperature range of 8, 15, 20, 25, 30, and 35 °C. Importantly, we kept the pH of the PDA medium consistent without any adjustments. The pH value of the PDA medium was measured at 5.6 ± 0.2 at 25 °C.

Following the temperature assessment, we proceeded to examine growth under varying pH levels. To achieve this, we adjusted the pH of the PDA plates to values of 4, 5, 6, 7, 8, and 9 with hydrochloric acid (Sigma-Aldrich, Germany) HCl (1N) and potassium hydroxide solution (Sigma-Aldrich, Germany) KOH (1N). PDA plates were inoculated with the conidia suspension as previously described and incubated at 25 °C for 15 days.

In both experiments described above, each experimental unit comprised a Petri dish, and we included four replicates for each treatment, totaling 24 samples for each experiment. For each Petri dish, we determined the diametral growth rate (mm day^−1^) by averaging the measurements at two perpendicular points using a digital caliper.

### Massive conidia production and germination of *B. bassiana* Bv065

To mass-produce *B. bassiana* Bv065, we followed the semisolid-state fermentation method detailed by Mejia et al. (2020) was used. The process consisted of the inoculation of aluminum trays filled with rice with a 2 mL suspension of 1 × 10^6^ conidia mL^−1^, followed by incubation at 25 °C for 7 days. After this period, the plastic film covering the trays was replaced with paper towels to promote sporulation and drying. Subsequently, the dried conidia were collected and sieved through a 150 µm mesh.

To assess conidial germination, 10 mg of dried conidia were suspended in 990 µL of 0.1% v/v Tween 80, and 100 µL of serial dilutions were inoculated into Petri dishes containing water agar supplemented with 0.1% w/v malt extract and 5 ppm benomyl. These Petri dishes were then incubated at 25 °C for 24 h, and germinated conidia were counted under a light microscope (ZEISS Axio Lab.A1, Oberkochen, Germany) at 40X magnification. Conidia with germinating tubes longer than their width or diameter were considered germinated, as described by Grijalba et al. (2018). Furthermore, conidial yield was estimated by counting conidia in serial dilutions using a Neubauer chamber (Boeco, Hamburg, Germany).

### Metabolic profiling of *B. bassiana* Bv065

The metabolic profile of *B. bassiana* Bv065 was assesed using the Biolog FF MicroPlates™ (Biolog, Hayward, USA), a microplated dish containing up to 95 carbon sources and a control well. To initiate this evaluation, we suspended fungal spores in 15 mL of FF-IF MicroPlate™ (filamentous fungi-inoculation fluid) broth (Biolog), adjusting them to a concentration of 1 × 10^6^ conidia mL^−1^. Subsequently, 100 µL of the conidia suspension were poured into each well of the microplates. The microplates were then subjected to an incubation period of 120 h at 25 °C, with constant agitation. This assay was conducted in triplicate to ensure robustness. We measured fungal growth at 750 nm and assimilation was assessed at 490 nm using a microplate reader (Cytation, Agilent Biotek, Santa Clara. USA), following the method described by Mchunu et al. in 2013.

### Enzymatic profile of *B. bassiana* Bv065

For the determination of lipase, chitinase, and protease activities originating from *B. bassiana* (Bv065), dried conidia were obtained from semisolid-state fermentation. The extraction of these enzymes involved suspending 50 mg of conidia in 1 mL of a 1% v/v Tween^®^ 80 (Sigma-Aldrich, Burlington) solution, followed by vortexing at a constant speed for 1 h. Afterward, we subjected the sample to centrifugation at 10,000 rpm for 10 min, and the resulting supernatant was collected for analysis. To quantify lipase activity, we followed the methodology outlined by Beys da Silva et al. (2010), and Glogauer et al. (2011). Chitinase activity assessment followed the protocol described by Santos et al. in 2017. In both cases, the reactions were carried out at 37 °C for 30 min, with absorbance measurements conducted at 400 nm using a Spectrophotometer (Cytation™ 3, Agilent Biotek, Santa Clara. USA). One unit of the enzyme was defined as the amount of enzyme that release 1 µmol of p-nitrophenol per minute at the conditions of the assay. To measure protease activity, we adhered to the procedure proposed by Cupp-Enyard (2008) was followed. Absorbance measurements were made at 660 nm using a Spectrophotometer (same as described above), and protease activity was expressed as the amount of enzyme needed to release 1 µmol of tyrosine per minute.

### Weevil collection and maintenance

Adult *R. palmarum* and *D. borassi* specimens were collected from oil palm (*Elaeis oleífera* x *Elaeis guineensis*) and peach palm (*Bactris gasipaes*) plantations using bucket traps as described by Lohr & Parra (2014). The traps contained a mixture of 300 g of sugar cane or pineapple, 6-methyl-2-hepten-4-ol (Rochat et al. 1991)and 4-Methyl-5-nonanol (Giblin-Davis et al. 1997) as aggregation pheromones, and 4.5 mL of ethyl acetate as attractants. The traps were checked weekly in peach palms and fortnightly in oil palms. The collected adults were subsequently examined in the laboratory to identify their species and sex based on morphological characteristics. Pairs of each species were housed in 500 mL plastic containers with sugar cane and allowed to acclimate for one week under laboratory conditions (12:12 LD photoperiod, 26 °C air temperature, and 60–70% relative humidity) before conducting experiments.

### Pathogenicity assessment of *B. bassiana* Bv065 against palm weevils

To evaluate the pathogenicity of *B. bassiana* Bv065, mortality experiments were carried out with two weevil species: *D. borassi* and *R. palmarum*. The experiments encompassed several treatments: (1) A suspension of *B. bassiana* Bv065 conidia at a concentration of 1 × 10^9^ conidia mL^−1^. (2) A commercial bioinsecticide containing the entomopathogenic fungus *Metarhizium anisopliae* at a concentration of 1 × 10^9^ conidia mL^−1^ (Metaran SC, Bioquirama, Rionegro, Colombia). (3) Another commercial bioinsecticide, which incorporated the entomopathogenic bacteria *Bacillus thuringiensis* at a concentration of 1 × 10^8^ UFC mL^−1^ (BTK, Bioquirama). (4) As a positive control, we employed the insecticide fipronil at a concentration of 0.6 mg active ingredient mL^−1^ (Trust 200 SC, PRECISAGRO SAS, Bogotá). This concentration was determined as the lethal dose required to induce mortality in 90% of *R. palmarum* in laboratory trials (Martínez et al. 2019). (5) For the negative control, mineral water with a 0.1% v/v Tween^®^ 80 solution was used.

At the beginning of the experiments, individual weevils were rinsed with clean mineral water and subsequently immersed for 30 s in their respective treatment solutions, all prepared in mineral water with 0.1% v/v Tween^®^ 80. Each treatment group comprised a pair of insects, consisting of one female and one male, housed in a clean 500 mL plastic container with two pieces of split sugarcane stalks for sustenance. The experiment was carried out under controlled laboratory conditions (26 ± 1°C and 60 ± 5% RH). The sugar cane was replaced every two weeks, and weevil mortality was monitored daily over a two-week period. The dead specimens were identified by their characteristic posture, lying on their side with retracted legs. These deceased weevils were then transferred to individual Petri dishes containing damp filter paper to confirm the presence of fungal sporulation.

### Data analysis

All statistical analyses were performed in R 4.1.1 (Core Team 2019) using RStudio (RStudio Team 2020). To investigate the impact of temperature on colony growth, we utilized a linear mixed-effects model (LMM) from the ‘lme4’ package (Bates et al. 2015). In this analysis, temperature and time (measured in days) were included as fixed effects. To account for the presence of repeated measurements, the experimental unit code was included as a random effect, addressing any temporal autocorrelation. Next, we conducted a similar LMM analysis to examine the influence of pH on colony growth. For this purpose, pH and time (day) were included as fixed effects, and the experimental unit code was again introduced as a random effect. In both cases, for the temperature and pH models, multiple comparisons were performed among treatments using the False Discovery Rate (FDR) method (Verhoeven et al. 2005) from the ‘emmeans’ package (Lenth et al. 2019).

To analyse the metabolic profile data, we computed the average fungal growth and substrate assimilation across all 96 substrates found in the microplates. These substrates were clustered into four groups using k-means (k = 4, based on preliminary data exploration of the within-cluster sum of squares). Following this, significant differences among these clusters were confirmed by conducting a Wilks’ lambda test within a multivariate analysis of variance (MANOVA). The Wilks test was used to determine pairwise differences between the clusters and adjusted the results for FDR using the ‘biotools’ package (Da Silva 2015). Furthermore, for the analysis of the enzymatic profile of *B. bassiana* Bv065 we applied descriptive statistics to the acquired measurements. Triplicate evaluations of chitinases, proteases, and lipases were used for the computation of both the mean and standard error for each distinct measurement.

Finally, to evaluate the pathogenicity of *B. bassiana* Bv065, a two-step analysis of the mortality data was conducted. Initially, the significance of the experimental factors and their interactions using a Cox Proportional Hazards Model were assessed, employing the survival package (Therneau 2016). This model included the experimental treatment, species identity, and sex as fixed effects. Subsequently, Kaplan–Meier Survival Curves was constructed for each weevil species separately using the survival package. These curves allowed us to compare the survival distributions of weevils subjected to different experimental treatments.

In all the models above, data distribution was identified using the library fitdistrplus (Delignette-Muller et al. 2019). The significance of terms in all the models was determined using Type-II analysis of variance (or Type-II ANOVA) with the ‘Anova’ function from the car library (Fox and Weisberg 2018). The library performance (Lüdecke et al. 2019) was used to inspect and plot the model diagnostics, and figures were made using the libraries sjPlot (Lüdecke 2016), survminer (Kassambara et al. 2017), and ggplot2 (Wickham 2016).

## Results

### Molecular identification of the isolated strain

The isolate Bv065 was identified as *B. bassiana* as evidenced by comparison with the GenBank sequences of ITS and TEF1-α fragments. For the former, 99.26% identity and an E-value of 0.0 were obtained when compared with the type material accession *B. bassiana* ARSEF (NR_111594.1). A 100% of identity with an E-value of 6 × 10^−149^ was observed when the Bv065 TEF1-α sequence was aligned with the sequence of accession *B. bassiana* isolate CVAD02 (P328925.1).

### Effect of pH and temperature on the growth of *B. bassiana* Bv065

**Fig. 1:**
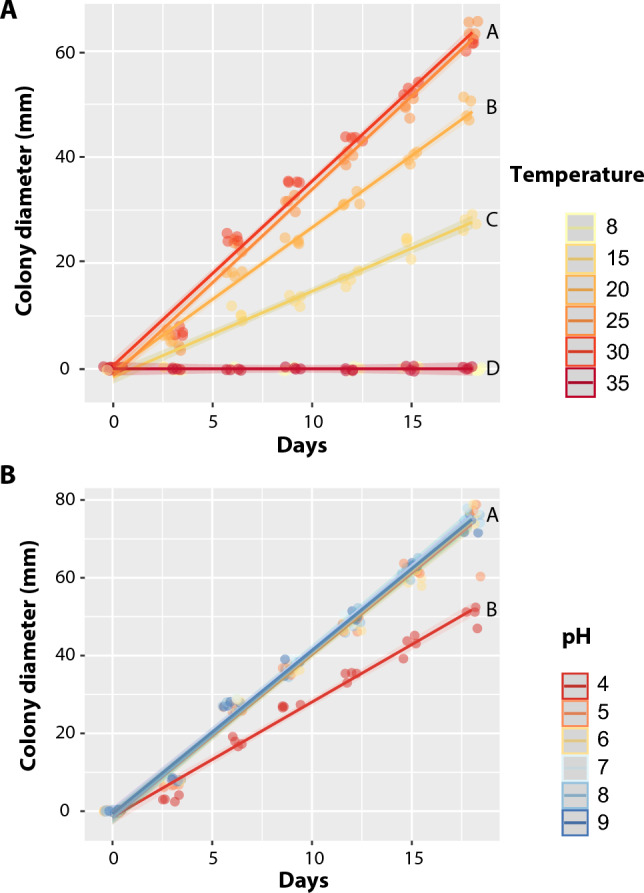
Influence of different incubation temperatures (**A**) and pH levels (**B**) on the growth of *B. bassiana* Bv065 over 15 days. Diametral growth was quantified by averaging measurements taken at two orthogonal points employing a digital caliper. The significance of differences is indicated by letters in accordance with pair-wise comparisons

The interaction between temperature and time significantly influenced the mycelial growth of the fungus *B. bassiana* Bv065 (LMM: χ^2^ = 3685.1, P < 0.001). At the end of the experiment at day 18, it became evident that Petri dishes exposed to temperatures of 8 and 35 °C exhibited no fungal growth. Among the temperatures ranging from 15 to 30 °C, noticeable variations in growth rates were observed, albeit following a linear pattern. The optimal temperature range for *B. bassiana* Bv065 growth was found to be between 25 and 30 °C, where the growth rate measured 3.47 ± 0.11 mm day^−1^ (x̄ ± SE). Importantly, there was no statistically significant difference between growth at 25 °C and 30 °C. Conversely, mycelial growth at 15 and 20 °C was restricted and notably slower compared to the rates observed at 25 and 30 °C (Fig. 1A, Table 1).

**Table 1:** Growth Rate (mm day^−1^) of *B. bassiana* Bv065 at varied temperature and pH conditions. Results are expressed as mean ± standard error

Growth rate of *B. bassiana* Bv065 (mm day^−1^)											
Temperature (°C)						pH					
8	15	20	25	30	35	4	5	6	7	8	9
0.00 D	1.54 ± 0.03 C	2.72 ± 0.06 B	3.56 ± 0.04 A	3.39 ± 0.02 A	0.00 D	2.81 ± 0.06 B	4.07 ± 0.24 A	4.26 ± 0.06 A	4.23 ± 0.05 A	4.21 ± 0.05 A	4.08 ± 0.07 A

Additionally, the pH levels and time interacted significantly, affecting the growth of *B. bassiana* Bv065 (LMM: χ^2^ = 183.21, P < 0.001). The *B. bassiana* isolate Bv065 exhibited robust mycelial growth across a broad pH range from 5 to 9, with a growth rate of 4.17 ± 0.23 mm day^−1^ (x̄ ± SE). However, growth was notably diminished at a pH of 4 in comparison to the other pH levels evaluated (Fig. 1B, Table 1).

### Massive conidia production and germination of *B. bassiana* Bv065

The *B. bassiana* Bv065 isolate yielded 6.8 × 10^9^ ± 1.7 × 10^8^ conidia g^−1^ of dry substrate (mean ± SE) following a 15-day semi-solid fermentation period. Notably, these conidia displayed a germination rate of 97 ± 1% within the first 24 h of incubation.

### Metabolic profile of *B. bassiana* Bv065

**Fig. 2:**
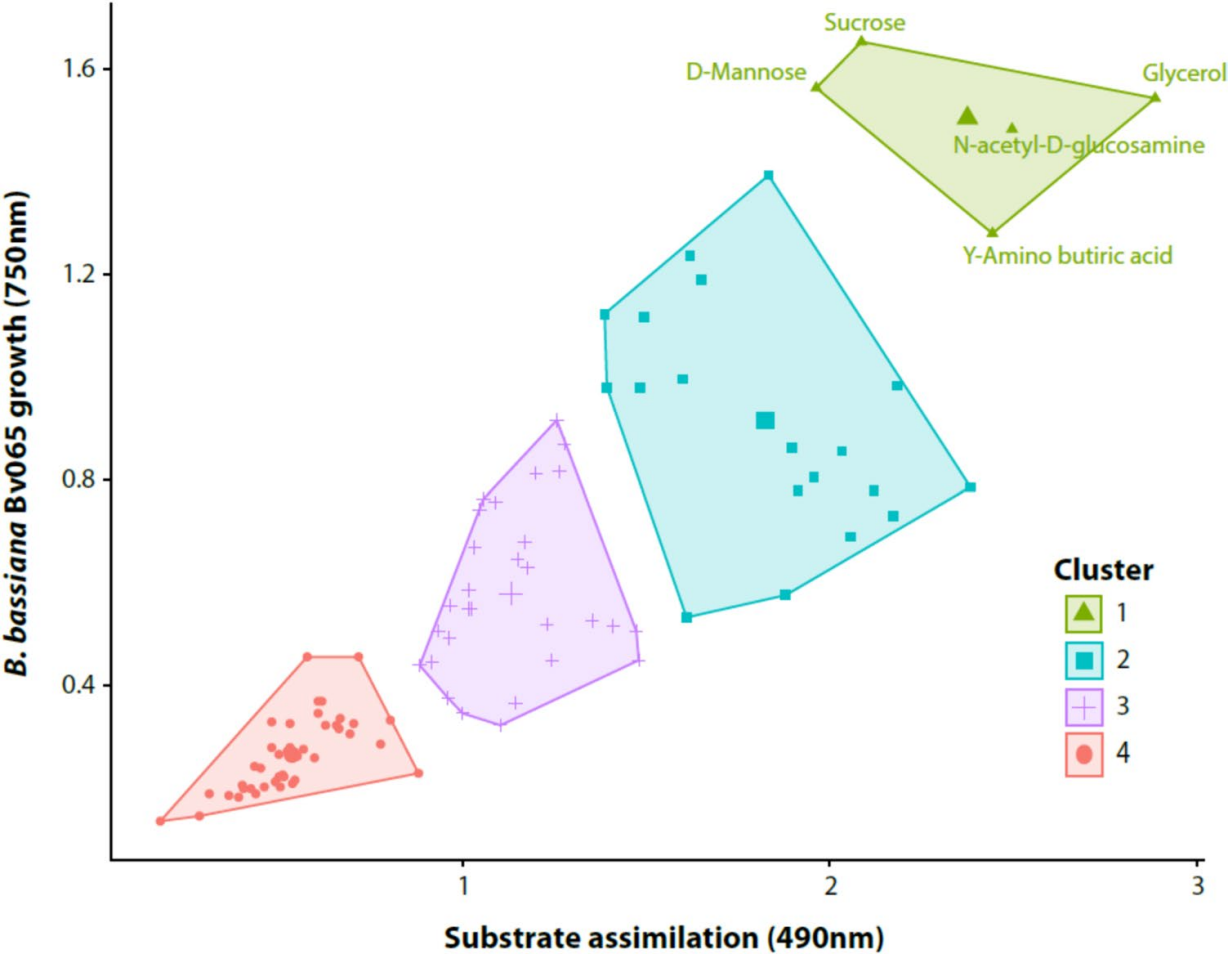
Impact of different carbon sources on *B. bassiana* Bv065 growth and substrate assimilation over 120 h. Growth assessments were conducted at 750 nm absorbance, while assimilation measurements were performed at 490 nm. Substrates were categorized into four distinct groups through the application of k-means clustering. Statistical analysis via MANOVA affirmed the presence of significant differences among these groups (P < 0.001). Detailed information on the substrates and their corresponding growth and assimilation values can be found in Table S1 in the supplementary material

Carbon source utilization profiles for *B. bassiana* Bv065 were analyzed using cluster analysis. The data generated for growth and substrate assimilation revealed four distinct substrate groups (MANOVA: F = 32.13, P < 0.001). The analysis showed that cluster I, composed of sucrose, D-Mannose, γ-amino-butyric acid, glycerol, and N-acetyl-D-glucosamine, exhibited the highest *B. bassiana* Bv065 growth and substrate assimilation. AT the cluster level, *B. bassiana* Bv065 achieved a growth of 1.50 ± 0.06 OD_750nm_ (mean ± SE) and a substrate assimilation of 2.37 ± 0.16 OD_490nm_ over a 120-h period (Fig. 2). Specifically, *B. bassiana* Bv065 achieved the greatest growth using sucrose, recording a value of 1.65 OD_750nm_ (mean ± SE) and an assimilation of 2.09 OD4_90nm_, followed by D-trehalose with a growth of 1.39 OD_750nm_ (mean ± SE) and an assimilation of 1.83 OD_490nm_. On the other hand, glycerol exhibited the highest assimilation with a value of 2.89 OD_490nm_ and a growth of 1.54 OD_750nm_.

Furthermore, cluster II exhibited a 24% decrease in assimilation and a 39% reduction in *B. bassiana* Bv065 growth compared to substrates from cluster I. Meanwhile, carbon sources from Groups III and IV showed a significant decrease of 50 and 60%, respectively, in both assimilation and fungal growth. Within these latter two groups, organic acids constitute most substrates at 86%, followed by amino groups at 80%, disaccharides at 67%, monosaccharides at 87%, polyalcohols at 90%, and all compounds classified as carboxylic acids, ester, and glycosides. The complete list of substrates within each group, along with precise values for *B. bassiana* Bv065 growth and substrate assimilation, can be found in Table S1 in the supplementary material.

In the context of each cluster, the five substrates grouped within cluster I, displaying the highest assimilation and growth values, are classified as amino acids, amino compounds, disaccharides, monosaccharides, and polyalcohols. Within cluster II, nine amino acids, two organic acids, three disaccharides, two monosaccharides, one polyalcohol, and two polysaccharides are found. Meanwhile, clusters III and IV encompass the remainder of the 72 evaluated substrates previously mentioned. In this study, it was observed that *B. bassiana* Bv065 achieved the highest assimilation and growth in 14 out of the 94 substrates comprising the Biolog FF™ plate.

In the comparative analysis of assimilation and growth results among different substrate groups, it was observed that the 15 amino acids exhibited the highest assimilation average, reaching a value of 1.73 ± 0.59 OD_490nm_. Meanwhile, the highest growth average was attained by the various disaccharides 0.79 ± 0.35 OD_750nm_ followed by the polysaccharides 0.70 ± 0.20 OD_750nm_. The amino acids with assimilation values exceeding 2.00 OD_490nm_ and a growth between 0.69 OD_750nm_ and 1.28 OD_750nm_ correspond to γ-amino-butyric acid, L-proline, L-glutamic acid, L-serine, N-acetyl-L-glutamic acid, glycyl-L-glutamic acid and L-alanyl-glycine. Disaccharides with assimilation exceeding 1.5 OD_490nm_ and greater growth ranging from 1.24 OD_750nm_ to 1.65 OD_750nm_ correspond to sucrose, D-Trehalose, and Maltose. Meanwhile, monosaccharides with assimilations surpassing 1.5 OD_490nm_ correspond to D-mannose, β-methyl-D-glucoside and α-D-glucose, with growth ranging between 1.12 OD_750nm_ and 1.56 OD_750nm_. Finally, *B. bassiana* Bv065 exhibited the lowest assimilation and growth values among compounds classified within the groups of dicarboxylic acids and esters, ranging between 0.48 OD_490nm_ to 0.51 OD_490nm_ for assimilation and 0.30 OD_750nm_ to 0.25 OD_750nm_ for growth, respectively.

### Enzimatic profile of *B. bassiana* Bv065

Proteases exhibited the highest activity among the enzymes measured, with levels reaching 3.69 ± 0.16 U mL^−1^ (mean ± SE). Conversely, chitinases showed a lower activity level, measuring 0.06 ± 0.01 U mL^−1^ (mean ± SE). Lipases, on the other hand, displayed no detectable activity, registering levels of 0 U mL^−1^ (mean ± SE).

### Pathogenicity assessment of *B. bassiana* Bv065 against palm weevils

The Cox Proportional Hazards Model revealed a significant interaction between the experimental treatment applied to the weevils and their species identity (CoxPH: χ2 = 22.71, P < 0.001). However, neither the weevils’ sex nor the interaction of sex with treatment or species identity showed statistical significance. Therefore, sex was not considered as a fixed effect in subsequent analyses.

**Fig. 3:**
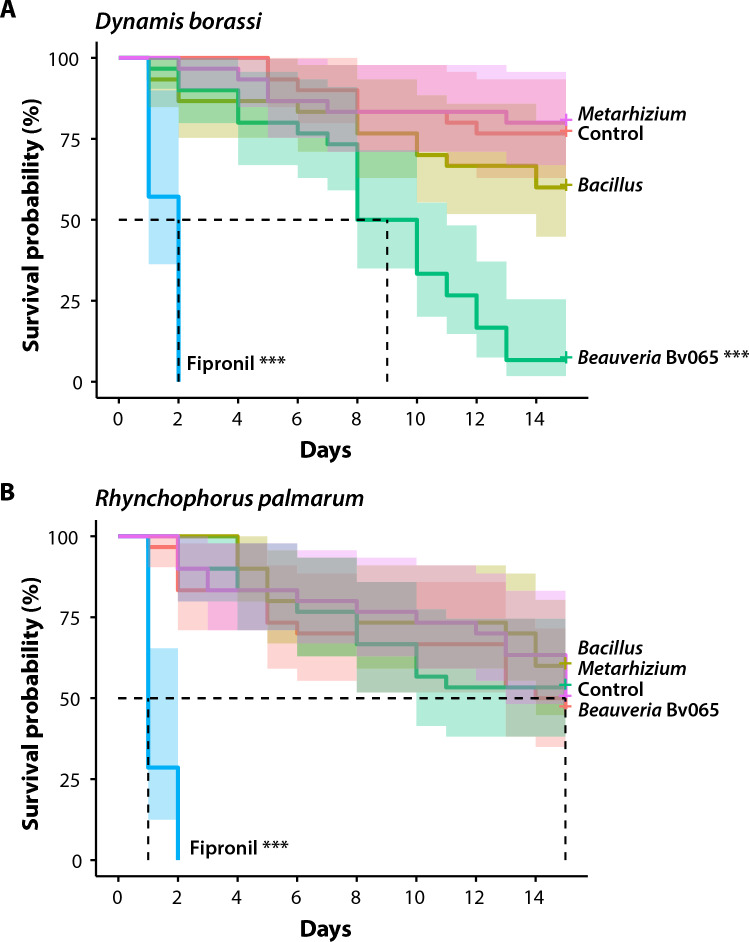
Evaluation of *B. bassiana* Bv065 pathogenicity. Survival analysis comparing the mortality of adult palm weevils of the species *D. borassi* (**A**) and *R. palmarum* (**B**) following treatment with *B. bassiana* Bv065, a negative control (water), a positive control (fipronil), and two commercial biological insecticides (*Bacillus* and *Metharizium*). *** indicates significant difference when compared with the positive control treatment

Furthermore, when examining survival distribution for each species separately, we observed that *D. borassi* weevils experienced 100% mortality when exposed to the positive control treatment with fipronil (P < 0.001). These insects perished within 48 h. Additionally, when *D. borassi* weevils were treated with *B. bassiana* Bv065, 50% of the test subjects died within nine days, and by day 15, approximately 94% of the insects had perished as well (P < 0.001). None of the other experimental treatments resulted in significant mortality for *D. borassi* (Fig. 3A). On the other hand, *R. palmarum* had its survival impacted solely by exposure to the control treatment, the insecticide fipronil, similar to *D. borassi*. In this case, all *R. palmarum* insects died within 48 h (P < 0.001). However, *R. palmarum* weevils did not exhibit significant susceptibility to treatment with *B. bassiana* Bv065 or any of the other experimental treatments (Fig. 3B).

## Discussion

This study represents an initial exploration into the isolation and characterisation of a strain of *B. bassiana* adept at managing a prevalent pest that impacts coconut and peach palm cultivation in neotropical regions, particularly targeting the weevil *D. borassi* (Gaviria et al. 2021; Hussain et al. 2022). Understanding the optimal growth conditions for a biological control agent is pivotal for crafting a highly effective and enduring biopesticide for field application. Factors such as temperature, humidity, radiation, and pH play pivotal roles in influencing the viability, persistence, and efficacy of entomopathogenic fungi throughout their production, storage, and application phases.

In our investigation, the *B. bassiana* Bv065 strain exhibited the highest growth rate within the temperature range of 25 to 30 °C, a range widely acknowledged for optimal growth among entomopathogenic fungi (Hallsworth and Magan 1999). Conversely, diminished growth at alternative temperatures could be attributed to phenomena such as protein denaturation and membrane disorganisation, thereby impeding the fungus’s growth and germination (Fernandes et al. 2009). Additionally, the *B. bassiana* Bv065 strain demonstrated optimal growth within a pH range of 6 to 8, indicating the strain’s proficiency in effectively regulating cytosolic pH (Hallsworth and Magan 1996; Gharibzahedi et al. 2014). Moreover, considering the typical pH range of insect cuticles, which range between 6 to 8, it is reasonable to assume that optimal growth at this pH leads to the expression of enzymes involved in the degradation and penetration of the insect cuticle by the fungus (Leger 1995).

Furthermore, we found that metabolites such as sucrose, D-mannose, γ-amino-butyric acid, glycerol, and N-acetyl-D-glucosamine play a major role in *B. bassiana* Bv065 strain metabolism. These metabolites were highly assimilated and induced the greatest growth of *B. bassiana* Bv065. These results align with those obtained by Canfora et al. (2017), who demonstrated that substrates such as N-acetyl-D-glucosamine, glycerol, D-trehalose, and γ-Amino-n-Butyric Acid, among others, are highly metabolised by *Beauveria* species. The affinity with N-acetyl-D-glucosamine reflects the relevance of *B. bassiana* Bv065 as an entomopathogen, as this metabolite is a component of the polysaccharide chitin found in the exoskeletons of arthropods (Le Guen et al. 2017). Additionally, the substrate for mass-producing fungus may influence important traits; for instance, it has been shown that sucrose and glycerol can influence the production of secondary metabolites such as polyketides and terpenes by entomopathogenic fungi, including the *Beauveria* genus (Zhang et al. 2020). Such secondary metabolites are known for their bioactive properties and their essential role in the pathogenicity of entomopathogenic fungi (Pedrini 2022; Toopaang et al. 2023).

The metabolic profile of *B. bassiana* has been identified as a major factor in the role of this fungal species as an entomopathogen. It has been established that *B. bassiana* is capable of growing in different environmental conditions due to its metabolic plasticity (Luo et al. 2015). Thus, the metabolic shift of *B. bassiana* is influenced by the availability and composition of carbon sources, impacting its growth, conidiation, stress tolerance, and pathogenicity (Mohamed et al. 2021; Gutiérrez Román et al. 2022). Additionally, the metabolic preferences of this genus for specific carbon sources are linked to its ability to repair DNA damage, improve growth, and modulate its lifecycle (Wang et al. 2019; Ren et al. 2021).

Moreover, the assimilation of substrates as a carbon source is highly dependent on the production of enzymes that catalyse their degradation. These enzymes are also considered crucial pathogenicity factors of entomopathogenic fungi (Golzan et al. 2023). In semi-solid fermentation, the Bv065 strain mainly produced proteases and chitinases, while lipases were not detected. Proteases play a crucial role in the pathogenicity of entomopathogenic fungi, aiding in the degradation of the insect cuticle and haemolymph, and facilitating invasion and growth within the host (Leger 1995; Khan et al. 2016). Similarly, chitinases may play a crucial role in this entomopathogenic fungus, particularly in cell wall morphogenesis and host-parasite interactions (Sahai and Manocha 1993). These enzymes are involved in the degradation of chitin, a major component of insect exoskeletons, allowing the fungus to penetrate and infect its host (Gooday et al. 1986). In the degradation process of the insect cuticle, N-acetyl-D-glucosamine is released (Liu et al. 2023), which in this study proved to be one of the substrates that significantly influenced fungal growth.

Regarding the pathogenicity of *B. bassiana* Bv065 strain against palm weevils, our results indicate that this strain is capable of infecting and killing adults of the species *D. borassi*, with greater effectiveness than other commercially available entomopathogens. The high mortality caused by this strain to individuals of *D. borassi* can be explained by the specificity of this fungus strain with this weevil species, as the original isolation was made after a *D. borassi* weevil naturally infected in field conditions. Conversely, the Bv065 strain was not significantly effective in causing lethal effects to the weevil species *R. palmarum*, which has shown susceptibility to other *B. bassiana* strains as reported in other studies (León-Martínez et al. 2019; Dalbon et al. 2024). Also, *B. bassiana* has been effective for controlling other palm weevil species like *R. ferrugineus* (Dembilio et al. 2010; Yang et al. 2023). Although the Bv065 strain may not cause significant lethal effects on palmarum, there may be sublethal effects of *B. bassiana* infection as suggested by previous studies. *B. bassiana* may cause a transgenerational effect on female weevils inoculated and that managed to survive infection. The offspring of such females (both in the egg and larvae stages) have lower survival rates and are more susceptible to *B. bassiana*. Additionally, surviving insects of the infection may act as potential vectors for the propagation of the entomopathogen and facilitate horizontal transmission of the entomopathogen (Lo Verde et al. 2015; León Martínez et al. 2019).

Since *D. borassi* and *R. palmarum* have been recognized as pests of economic importance for the crops of *Bactris gasipaes* and *Cocos nucifera* (Bautista-Giraldo et al. 2020; Gaviria et al. 2021; Gutiérrez et al. 2023), the results obtained with the Bv065 strain show great potential for its use in the field, where different dissemination strategies can be employed such as trunk applications (Güerri-Agulló et al. 2010), passage traps for the inoculation of adults (Dembilio et al. 2018), release of contaminated males (León Martínez et al. 2019); where the control objective is the adults. Additionally, exploratory studies have shown the potential use of *B. bassiana* in endotherapy (i.e., trunk injection) for the control of weevil larvae inside the palms (Sutanto et al. 2023; Husain et al. 2024).

Overall, the *B. bassiana* Bv065 strain exhibited distinctive traits across diverse conditions, reflecting the natural habitat of palm weevils in Colombia. The optimal growth range for *B. bassiana* Bv065 in terms of temperature and pH provides the basis for optimizing mass production and identifying bottlenecks for use in field conditions. Furthermore, the metabolic profiling assessment of *B. bassiana* Bv065 enabled the identification of different carbon sources that would increase the growth of this entomopathogenic fungus. This serves the development of a culture medium conducive to high fungal growth, sporulation, and biological activity. Additionally, it facilitated the identification of substances viable for use in the large-scale production process and subsequent formulation of a bio-pesticide, utilizing this strain as the primary active ingredient. Finally, the promising preliminary pathogenicity assessments show that this strain is effective for the control of the pervasive palm weevil *D. borassi*.

### Supplementary Information

Below is the link to the electronic supplementary material.

Supplementary file 1 (XLSX 20 KB)

## Data Availability

The data that support the findings of this study are available on request from the corresponding author, YG
